# Promoting wellbeing and improving access to mental health care through community champions in rural India: the *Atmiyata* intervention approach

**DOI:** 10.1186/s13033-016-0113-3

**Published:** 2017-01-04

**Authors:** Laura Shields-Zeeman, Soumitra Pathare, Bethany Hipple Walters, Nandita Kapadia-Kundu, Kaustubh Joag

**Affiliations:** 1 Trimbos International Department, Trimbos Institute , Utrecht, The Netherlands; 2 Centre for Mental Health Law and Policy, Indian Law Society, Pune, India

**Keywords:** Community mental health, Mhealth, Community-based intervention, Evidence-based intervention, India, Low and middle-income country

## Abstract

**Background:**

There are limited accounts of community-based interventions for reducing distress or providing support for people with common mental disorders (CMDs) in low and middle-income countries. The recently implemented *Atmiyata* programme is one such community-based mental health intervention focused on promoting wellness and reducing distress through community volunteers in a rural area in the state of Maharashtra, India.

**Case presentation:**

This case study describes the content and the process of implementation of *Atmiyata* and how community volunteers were trained to become *Atmiyata* champions and mitras (*friends*). The *Atmiyata* programme trained Atmiyata champions to provide support and basic counselling to community members with common mental health disorders, facilitate access to mental health care and social benefits, improve community awareness of mental health issues, and to promote well-being. Challenges to implementation included logistical challenges (difficult terrain and weather conditions at the implementation site), content-related challenges (securing social welfare benefits for people with CMDs), and partnership challenges (turnover of public health workers involved in referral chain, resistance from public sector mental health specialists).

**Conclusions:**

The case study serves as an example for how such a model can be sustained over time at low cost. The next steps of the programme include evaluation of the impact of the *Atmiyata* intervention through a pre-post study and adapting the intervention for further scale-up in other settings in India.

## Background

Mental ill health is a substantial public health burden in India. Approximately 70 million people in India experience some form of mental illness, of which many have limited to no access to mental health support and treatment [1–5]. Of those experiencing mental health problems, approximately 20% of the Indian population is affected by common mental health disorders (CMDs) such as anxiety and depression. Disorders such as these are often under-detected and undertreated due to a variety of factors [6]. People with mental health problems often face discrimination in their communities [7–11], which can reduce willingness to seek help from mental health care providers. Supply side factors, such as the paucity of trained mental health professionals in India, means that there are insufficient human resources to address the burden of CMD in the community [12], particularly in rural areas [13]. While primary care practitioners in rural communities can provide mental health care, they typically lack the skills needed beyond very basic care [14]; this results in a growing disparity to access of any form of mental health care in rural India, despite the evident need [15, 16]. The limited number of mental health care providers and the limited services available, combined with the fear of discrimination, contribute to the notable treatment gap for mental illness in India [17–19].

### Mental health care in Indian villages

At the village level in India, mental health care is primarily provided through community mental health workers and non-specialised health workers [20, 21]. Several programmes have been developed in recent years to build the capacity of community health workers and/or primary care level health workers with the aim of increasing their uptake of mental health tasks [19, 22]. Research has demonstrated the efficacy of such initiatives in India as well as in other parts of South Asia [22–25]. Similarly, the public health worker model, as supported by the District Mental Health Programme, has shown impact in small pilot projects and research projects, but these projects have not been scaled up. It is possible that these interventions and this public health worker model have not been scaled up due to concern that formal health workers are already burdened with other health care tasks (such as child and maternal health, vaccination tasks), leaving limited time for mental health. In recent years, service delivery models for mental health in low and middle-income countries have focused on task-shifting, which is the process of delegating tasks to less specialised health workers or to a health worker with different education or training [20, 23, 26]. In mental health, task-shifting has been done primarily through lay health workers providing counselling or treatment for mental health problems [22, 26–29]. However, such service delivery models face challenges related to capacity and to the lack of sustainable financial mechanisms or incentives for lay health workers, particularly in the context of the currently overburdened public health system in India [30–33]. To address concerns related to professional capacity, financing, and sustainability, one solution may be to use community members to identify and support their fellow community members in improving wellbeing in rural India and narrow the treatment gap.

In many villages across India, community resource groups already exist in the form of self-help groups (SHG’s) and farmer’s clubs (FC’s). In rural parts of India, self-help groups typically consist of a group of 15–20 women that voluntarily come together for peer support and focus on empowerment, cultivating entrepreneurial spirit, engaging in opportunities for economic development, and participating in local governance structures [34]. These community resource groups are widespread across the country; in the state of Maharashtra alone, there are over 200,000 self-help groups. Farmer’s clubs are grassroots-level forums consisting of men, often farmers or involved in agricultural work that come together for similar purposes to SHGs. Such community groups typically have one elected group leader.

Using community groups to discuss health topics such as pregnancy has yielded positive outcomes on maternal health indicators in Eastern India and Nepal [35–37], showing reductions on maternal and neonatal mortality rates and on depression rates. Participating in either a self-help group or a farmer’s club is both socially and culturally acceptable; existing community organisations therefore may offer a unique opportunity for identifying and supporting community members in distress or affected by a mental health problem in an acceptable, open, and supportive space in the community. As both self-help groups and farmer’s clubs are currently active in many villages, building the capacity of these groups for detecting, supporting, and referring people in distress or affected by a mental health problem has a high potential for creating more sustainable and locally available options for mental health support in an area with limited formal treatment options.

## Case presentation

The ultimate impact which the Atmiyata programme aimed to develop community-based mental health and social care pathways to reduce the treatment gap and contribute to achievement of a higher quality of life for people with CMDs and severe mental illness in Atmiyata villages. To this end, the primary outcomes of the Atmiyata programme are increased detection of people with CMDs and SMI, increased access to mental health supports and social entitlements, and increased awareness of wellness and distress. The *Atmiyata* programme consisted of a community-led intervention that aimed to promote wellbeing and reduce the burden of mental illness in the community through training a core group of community members to become Atmiyata champions. The role of these champions was detecting mental health problems, providing basic treatment and support, and referring those mental health problems in need of further treatment to mental health professionals. Atmiyata *mitras* were a separate but complementary group of community members receiving comparatively less training, and were trained on two core competences: to identify distress among village members and refer them to Atmiyata champions for further support, and to provide information about distress and wellbeing to community members. A parallel aim of the programme was to address the economic deprivation of the community by training Atmiyata champions in facilitating access to social care benefits offered by the government, which provide a small financial contribution to households of participants every month. Thus the *Atmiyata* intervention was designed to address poverty, which plays a role in mental health problems [38]. The intervention was founded on building on existing community strengths and resources with the ultimate aim to strengthen overall community well-being.

The development and evaluation were funded by Grand Challenges Canada (Grant number: 0327-04) as a pilot programme in the state of Maharashtra, India over a period of 24 months. The programme was implemented in partnership with a local non-governmental organisation, BAIF Development Research Foundation. BAIF has worked in the study area for the past 15 years with local SHGs. Field supervisors for *Atmiyata* were BAIF employees (ranging from Ayurvedic doctors to social workers) who had experience working with self-help groups in the past. The supervisors played an important role in the selection, monitoring, and supervision of *Atmiyata champions*.

The *Atmiyata* approach complements other community-based mental health projects in South Asia [22, 24, 39] but has distinct differences from other community-based health worker or lay health worker models. First, within the context of the World Health Organization’s pyramid of service provision for mental health care [40], projects have focused on training primary health care staff or other health professionals to provide mental health care at the community level. The *Atmiyata* approach, however, intervenes one level below on the pyramid of mental health services in the domain of informal care and trains ordinary community members in providing basic mental health support. Second, the *Atmiyata* intervention uses volunteers as opposed to introducing new health workers or paying lay workers. Third, *Atmiyata* harnesses community resources through an approach based on social capital [41, 42] and is focused on low-intensity psychosocial interventions, [43] as opposed to adopting a medical model for mental health problems.

**Fig. 1:**
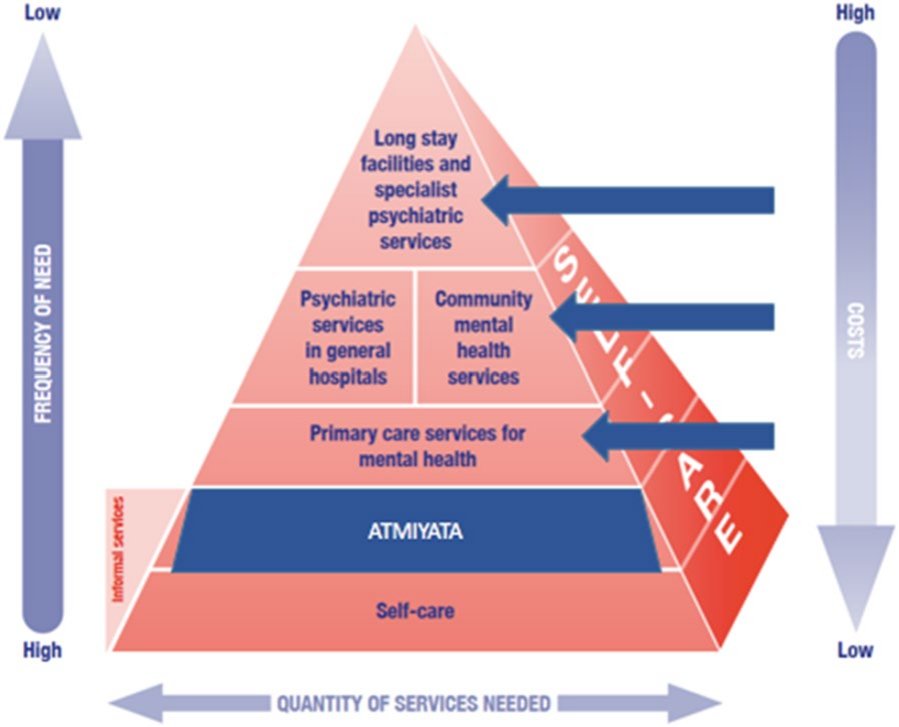
Adaptation of WHO service organization pyramid detailing the optimal mix of mental health services [40] for the *Atmiyata* project

The word *Atmiyata* means shared compassion in Marathi, the local language in the Indian state of Maharashtra. Shared compassion serves as the core tenet of this intervention and is based, in part, on the ancient Indian theory of communication, Sadharanikaran [44–46]. In order to promote sustainability, to reduce costs, and to work around the lack of highly trained mental health care providers, *Atmiyata* focused on the lower steps of the pyramid of care (informal community care, Fig. 1) [40]. Rather than paying community members to participate, the project recruited volunteers from self-help groups and farmer’s clubs in the region. Working with volunteers enhanced the potential for sustainability as volunteers were embedded in project villages, reduced cost of the intervention, and worked with those who were motivated to contribute to community wellbeing and to strengthening community cohesion.

Interventions provided by *Atmiyata* champions addressed both social and (mental) health care needs. These include evidence-based low-intensity counselling techniques such as active listening, problem solving and behavioural activation, as well as facilitating access to social benefits (e.g. disability benefits, unemployment benefits). The *Atmiyata* programme also established a care pathway for mental health treatment; this care pathway begins with support provided through a two-tiered network of volunteer community mobilisers. *Atmiyata mitras*, who received less training than the champions, provided information to community members on general wellbeing, healthy lifestyle management, and referred people they assess may be in distress or have mental health issues to champions. *Atmiyata* champions provided low intensity counselling and referrals to community members.

The programme trained *Atmiyata champions* in using the referral chain established and put in place by the project team. This involved frequent communication with the psychiatrist working at the district level. Champions were trained on how to recognise whether a community member required a referral based on the signs and symptoms of a mental disorder and when to assess if care needs were more severe. When community members are assessed by champions to be in a more severe phase of their illness and/or have more complex care needs, the *Atmiyata champions* referred and accompanied them to the district psychiatrist and district mental health centre for more specialised mental health care.

The philosophy behind the *Atmiyata* approach lies in its emphasis on tapping into social capital and encouraging community members to help their fellow community members through supportive techniques to enhance well-being, mental health and improve socioeconomic conditions. In this way, Atmiyata champions enhance their own social network and status within their communities as a result of contributing to the greater wellbeing of the community. The project’s focus on psychosocial aspects and wellbeing as opposed to a medical approach was also reflected in the project’s terminology. The project actively discouraged the use of mental health terminology and instead used terms like “stress of day-to-day living” and “distress”. The core focus within the project team was on building community capacity to solve problems and reduce distress as opposed to adopting a highly medicalised, top-down psychiatrist-driven approach. In addition, *Atmiyata* was designed to embed a mental health interventions within existing community development programmes in the region in order to create a more integrated programme that carries less stigma than standalone mental health programmes.

### Target groups

The *ATMIYATA* intervention targeted two populations experiencing varying levels of distress:

1. People with emotional stress and/or common mental health problems: The project trained *Atmiyata champions t*o refer and provide ongoing support and address both health and social needs, and trained *Atmiyata mitras* in 41 villages to detect symptoms and refer community members to *Atmiyata champions.* These target groups receive support from *Atmiyata champions* to better handle and cope with stressful situations. This level of support served as preventative measure for preventing the exacerbation of anxiety or depressive symptoms, as well as promote healthy lifestyle behaviours (e.g. sleep hygiene, exercises, yoga, behavioural interventions such as behavioural activation, and problem solving approaches—motivating people to work, go to group meetings, or attend social functions). All people detected to have a CMD received 4–6 sessions of low intensity counselling.
2. People with severe mental illness: People with severe mental illness were referred to the district hospital level for specialised assessment and care provided by a psychiatrist. *Atmiyata champions* accompanied clients on their first visit to facilitate the process and later ensured that the person maintained follow up with the district mental health services as recommended. *Atmiyata champions* also supported all target groups in accessing and obtaining social benefits available in the state related to pension, employment, and other livelihood schemes for people with mental health problems and their family members.

### Programme population

The programme was implemented in 41 villages, targeting approximately 14,000 adults in the Peth block (*geographical district subdivision)* of Nashik district in the state of Maharashtra. This block was chosen based on two reasons: first, census indicators from the Government of India showed significant socio-economic deprivation and the area was in need of locally available treatment options, and for the possibility of having a local field office. Second, as the local implementation partners of the project had a base in Peth block and the city of Nashik as well as a track record of ongoing community development and livelihood programmes in the area. The project villages are situated approximately 50 km away (in difficult transportation conditions) from the city of Nashik. Nashik district has only one public mental health facility which is a district general hospital, consisting of an outpatient clinic and ten inpatient beds. In terms of the demographic profile of the population, the overwhelming majority of the Peth population (96%) belongs to Scheduled Tribes (*tribal populations*) who face social and economic deprivation. To illustrate, 68% of households are below the official poverty line, and the literacy rate is 60% (below the state average of 72%) and only 1.16% of agricultural land is irrigated. The majority (52.4%) of villages in Peth block do have access to any public health facility, but only 7 out of 145 villages in the block have a primary health centre (PHC) and there is only one rural hospital catering to the entire population of the block.

### Intervention development

A first step in developing the *Atmiyata* intervention was to carry out a needs assessment in the 41 study villages; this was done through 10 focus group discussions and 12 in-depth interviews in order to understand community needs for mental health and social care. In addition to mapping needs through the focus groups, interviews also explored existing strategies used locally when a community member was in distress or had a mental health problem. The results from the interviews and focus group discussions helped shape the content of the intervention, particularly the content and angle for the development of the community films. After the needs assessment, the project developed and implemented a village-level mapping tool called the *Mohalla Mapping Tool*. The aim of this tool was to develop a graphical representation through village maps of who the *Atmiyata champions* were and where they were located, as well as map the households in each villages experiencing distress or a possible mental health problem. This process was done by the BAIF field supervisors together with the Atmiyata champions. Champions would draw on the paper-based maps where in the village they had identified people in distress, and what kinds of problems identified community members were facing. By doing this, not only could the project see how many people in each respective village were in need of support by the *Atmiyata champion* but also allowed the project to operationalise the concept of intervention coverage, through use of the maps, to identify whether the intervention was reaching all parts of the villages which the *Atmiyata champions or mitras* see community members. These maps were subsequently digitalized to provide estimates of the number of people identified with problems in each of the project villages, as well as the number of people who accessed some form of care.

### Implementing care pathways

As *Atmiyata champions and mitras* were trained to detect and help people experiencing stress or CMDs, it was essential for Atmiyata champions to remain motivated and committed to the work that they do to feel supported by a broader network of health and social care professionals should a person’s care needs exceed their competencies. The local field staff team, consisting of two psychiatrists, a community public health expert, and non-governmental workers familiar with the project sites, worked closely with district and local level public health authorities to build a referral pathway for those who required more specialised care (e.g. severe depressive episode, psychotic episode, risk of suicide). Addresses and names of psychiatrists located at the district hospital in Nashik, and primary health care centre in Karanjali were provided to champions on paper as well as in the app. When the champion identified a person with a moderate or severe CMD, or someone they suspect might show signs of a severe mental disorder, they worked directly with the person in need and their families to go to the District Hospital. In the event the person was unable to financially afford transport, the champion spoke to village leaders (such as elected head of the local gram Panchayat, which is the local self-government organisation at the village or small town level) who then arranged transport. Champions accompanied the person to the district hospital for their first visit and liaised with the district psychiatrist or doctor which helped to ensure a smooth consultation, as prior linkages made by the project team between local psychiatrists and champions meant that care was more prompt than prior to implementation without such referral pathways in place. People who experienced somatic problems as well as CMDs often went to the Primary Care Centre in Karanjali and were accompanied by a Champion.

Various tools and materials were needed to implement the *Atmiyata* intervention in communities. These tools and materials were developed to meet the needs of the community members and *Atmiyata champions* during and after the project period. These included training materials, educational films, an app for showing basic signs and symptoms of mental health issues as well as a platform for viewing the films, and a method of mapping the intervention to the needs of the community. The paper-based training manual for the champions was extensive and included an introduction to common mental and physical health complaints, information and guidance on how to start a conversation for those who may be experiencing distress, tips on how to conduct a mental health support group in the community, training on how to do active listening, problem solving and behaviour activation, and guidance on when and how to refer those with more severe symptoms to additional services. It also included cue cards and symptom cards which were previously used in the Thinking Healthy Programme Manual [39]. The manual was inspired by and based on previous manuals for non-specialised health workers in India working in mental health [39] and input from the project team’s previous work in India and in other countries. The training manual was first piloted among a selection of champions, adapted to include more examples and case descriptions, and then used in training for *Atmiyata champions*. Each *Atmiyata champion* was provided with quick reference information on referral points of health and social care professionals within the care pathway set in place in the project.

### Development of films

Seven films were created and used as training tools for *Atmiyata champions* and mitras to better understand mental health concepts and the importance of mental health. Training films were structured similar to chat shows, with project staff and locally recognised personnel from non-governmental organizations answering questions and concerns that the *Atmiyata champions* and mitras may have had, identified by the project team during initial focus groups and interviews with the SHG and FC members in the project villages. These films were uploaded on the smart phones provided to the *Atmiyata champions* and could be viewed offline any time, allowing the mitras and champions to refresh their knowledge and skills on an as-needed basis.

To facilitate dialogue among community members on distress and mental health, the *Atmiyata* intervention developed and deployed four community films to enhance community awareness. The formative research carried out by the project revealed several common distressing social situations experienced in every village: domestic violence, alcoholism, unemployment and spousal conflict. Four community films were developed on each of these themes in the local language (Marathi) with local actors. A director, cameraman and the producer formed the production team while a technical team of two psychiatrists and a behaviour change specialist developed the scripts for the films through a round of drafts. In addition, music was identified in the formative research as an important element of community life in the project villages; therefore, the films used background music to resonate more closely with community members viewing them.

Once the films were developed, they were loaded on smartphones and disseminated to Champions. Champions held community group meetings as part of their work as SHG or FC leader. During these meetings, the films were shown on the smartphone. To support discussion, the films have designated pause points; these pause points (which are in black and white, with a frozen frame) created the room and opportunity for the champion to ask the group about issues that they may face that are similar to those shown in the films. The project team provided these questions and discussion points to the Champions. The films are available for free to download on YouTube [47–50].Since the films were downloaded and available offline for viewing at any time, they could also be used during one-on-one conversations between the champions and members of the community. The films are still available and are still being used in Peth. In addition, when champions were speaking with clients, they often showed the films first to the clients as a starting point for dialogue.

### Development of an Atmiyata app

**Fig. 2:**
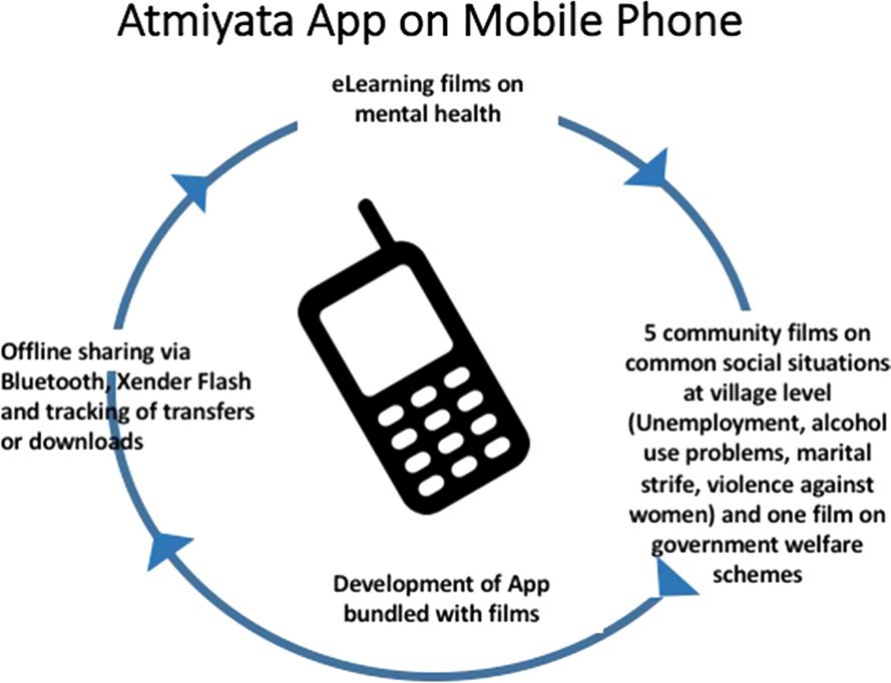
Overview of the components of the *Atmiyata* app for use on smartphones

The *Atmiyata* app is a free Android-based application, developed by a local technology company commissioned by the project team (Fig. 2). The project created two versions of the application, one for *Atmiyata Champions* and one that could be used for the general public/community members. *Atmiyata Champions and mitras* spread the word about the app to community members and where to access it. Both versions of the app are simple and have three screens: one screen for a list of films, second screen with a Bluetooth button for sharing the app with others, and the third screen with information about the project and contact details of BAIF (the field-based organization) for concerns or emergencies. The majority of functionalities of the app for the general public and community members could be used by people who were not literate.

The app version for Champions had training and community films uploaded within it, whereas the app version for the general public/community members had only the community films. The app version for champions had 8–10 questions included after the training films testing knowledge of mental health topics covered in the films. These questions had to be answered after every film viewing, and this was a process and prompt which was built into the app. Data from these questionnaires were collected and converted into a monthly report showing the answers to the questions, how many times the films have been viewed, and which films were viewed. In the event that the app was shared by Bluetooth to another person, before installing the app a consent form appeared on the smartphone screen which set out terms and conditions which needed to be read and accepted by the user. Clicking on *yes* generates an SMS text message which comes to one of the project team’s mobile phones, allowing for the project team to ascertain how many times the app had been shared with others.

### Building local capacity in mental health and wellbeing support

Training local community members to serve as a resource for community-based support was a key component of the *Atmiyata* intervention. Initially, 65 candidates were selected by the project team for participating in the project; however, 8 candidates dropped out of the training programme due to not being able to sustain work without payment. Two new candidates were subsequently identified by the project team and in total, 59 community facilitators (referred to as *Atmiyata champions*) and 264 community supporters (*Atmiyata Mitras*) were trained. The intervention created a two-tiered system of community volunteers; these volunteers served as a strong community resource for people within the project catchment area of Peth. The idea of the *Mitras* emerged during implementation as an additional support to the *Champions* to identify people with mental health problems.

Based on information learned during the needs assessment interviews as well as information and experiences of staff from the non-governmental organization partner in the project, the project developed selection criteria for *Atmiyata champions*. Criteria for selection included: being a leader of a SHG or FC, commitment to working on a voluntary basis, good communication skills, demonstrated knowledge and insight about their own community and culture, sense of pride in helping others and willingness to spare time for training and work. It was not mandatory to have a certain level of literacy skills but the project gave preference to those who had basic literacy (could read and write). The *Champions* did not receive any monetary compensation, but they were encouraged to see their capacity building, award certificates and trophies at the end of the training, and having a smartphone for their use as in-kind compensation for their effort. *Champions* were allocated any designated geographical area but were asked to cover their neighborhood in their villages.

Training of the *Atmiyata champions* was led by the Principal Investigator (Psychiatrist) and assisted by other project members (psychiatrist and behavior change specialists) and by BAIF employees with experience in social care and rural development. The training structure consists of seven days of core content-related training in community centres. Training was both a mix of theory sessions and practical sessions, ranging from classroom-based lectures, films and interactive discussion to role-plays, how to use symptom cards, and practicing dialogue and counselling sessions with community members. Champions also received training on problem solving and behavioural activation. The training for champions included a close review of the programme manual. Referral pathways (for more severe cases) and how to facilitate social welfare benefits for community members were also core topics of the training for champions. Additional training modules were provided to *Atmiyata champions* for several purposes. Champions were provided with mobile smartphones by the project. After receiving the smartphones, champions were provided one day of training on how to use the mobile smartphones and one day of training for how to use the films as a tool to facilitate community groups. Additional 4-h coaching sessions were provided in person to champions on low intensity counselling, as well as a 4-h session on how to use the Mohalla Mapping tool in the field to map distress in their communities. Both additional training sessions were provided either by clinicians or by field supervisors. This training was enhanced by follow-up and supervision site visits every two weeks. The structure of these follow-up and supervision site visits involved meeting champions and discussing challenges or concerns they encountered in their villages. The visits also entailed collecting monitoring data that had been collected since the last data point, participate in and/or lead a group meeting with *Atmiyata Mitras* or with a self-help group or farmer club, and plan the next on-site visit. The supervisor would also often accompany the *Atmiyata champion* to visit people with mental health problems and their family members. In addition, every alternate month the project team held troubleshooting sessions with the champions to address emerging needs.

*Atmiyata Mitras* received one full day of training. Mitras were paired with *Atmiyata Champions* (with a ratio of 5:1 mitras to champions) to combine community-level efforts and to receive additional guidance and support from Champions. The aim of creating this group of community supporters was to increase the coverage of support that community champions and supporters can reach in their villages; to intensify efforts to identify community members in distress and with severe mental health problems; and to spread knowledge about wellness and social benefits, particularly for vulnerable populations and amplify the work of the champions.

### Implementation of Atmiyata

To assess the impact of the *Atmiyata* intervention at the population level, the project team employed a quasi-experimental pre-post control group design. A pre-post survey was developed and carried out in 2014–2015 to screen for CMDs, assess overall community wellbeing, economic burden of care, social capital, and substance use.

In total 59 *Atmiyata Champions* and 264 *Atmiyata mitras* received the training and received follow-up mentoring and support available from the project team while working in the field. After the training of *Atmiyata champions* and *mitras* was complete and the mobile films and app were ready, the core component of the project began. For each of the 41 villages in the project, 1 or 2 champions were assigned to each village and 4–5 *Atmiyata mitras* were assigned to each village. *Atmiyata champions* were asked to detect distress and mental health problems, and when necessary, conduct 4–6 sessions of low intensity psychological interventions. If required or if there was no response to the psychological interventions provided, the *Atmiyata champion* would refer the person to the district hospital for more specialised care. Regardless of severity, people seeking help from *Atmiyata champions* were encouraged to access social entitlements and supports to facilitate social problems such as poverty and unemployment. *Atmiyata mitras* had the task of identifying people in their villages who might require support and to serve as the linking pin between the community members and the Champions.

### Challenges in implementation

There were several unexpected challenges in project implementation that are important considerations for future implementation of *Atmiyata*. First, the location of the project site made data collection, field visits, and evaluation difficult. The project area had turbulent weather which compromised travel routes. In addition, being a tribal area surrounded by difficult terrain, connection with other districts was difficult and mobile phone connectivity could be poor. This challenge was solved through careful planning and ensuring that to the extent possible, local field staff had close collaboration with *Atmiyata champions* through text messages, by phone, or through in-person supervision visits.

Content-related challenges were encountered particularly with securing social welfare benefits for community members. Initially the project envisaged obtaining disability certificates for people with mental health problems, which in India are issued by psychiatrists. However, the public health psychiatrist in the district was reluctant to issue disability certificates due to the belief that disability is a permanent state and such a certificate could not be issued to someone who had been experiencing symptoms for a temporary period of time (e.g. several months). Instead, the project then focused on securing other social welfare benefits such as securing pension allowance. In addition, while many health professionals were cooperative within the referral pathway, the primary health care medical officer refused to consult people with severe mental illness during follow-up visits. This meant that people with a severe mental disorder had to travel 50 kilometres to the nearest city to get medication, which impacts medication adherence on a long-term basis. In addition, the district psychiatrist changed during project implementation, which meant that the project needed to again build rapport with the new psychiatrist. High turnover of staff in India exists and therefore it is important to have a contingency plan in place for how to approach new staff or decision-makers.

## Conclusions

This paper presents the *Atmiyata* programme, which has been implemented in a rural part of the state of Maharashtra, India from 2013 to 2015 as a case study for a potential model for community-based mental health care for supporting people in distress, experiencing CMDs or experiencing severe mental disorders in low and middle-income countries.

Several potential good practice points for other programmes and community-based mental health interventions can be derived from the project design and implementation approach of Atmiyata. First, liaison and engagement with the public health system is crucial. Operating outside the public health system (i.e. through partnership with private practitioners) is not a sustainable service delivery mode, particularly at the village level where the most accessible and affordable health services and resources are through the public health system. Second, a clear care pathway with clear roles and responsibilities at each level of care (community, primary, secondary and tertiary) is essential to have the supports necessary for *Atmiyata champions and mitras* to feel confident in carrying out their tasks. Important care pathway considerations for future community-led interventions are establishing formal collaboration with a psychiatrist and/or a psychiatric nurse at a district hospital, if the project takes place at the village level. In the Atmiyata programme, having frequent communication and engagement with the district psychiatrist helped in securing consultations for people referred by the Atmiyata champion and also helped in identifying more effective treatment options. In addition, establishing common ways of working with district-level mental health staff is important. In some instances, this may be an agreed upon schedule in place where district hospital staff visit rural hospitals closer to the villages for clients who cannot travel to the district hospital. Consideration of whether additional human resources can be allocated to a rural hospital to offer care closer to people’s homes is also important. This could include placing a trained counsellor at the rural hospital, which was been done in Goa [22], or by leveraging human resources within the District Mental Health Programme, which allocates one psychiatrist to each district in India which is closer to villages than a rural hospital [12]. Second, using digital tools such as low-cost smartphones with easy to use apps or films can serve as an incentive for community volunteers as well as accelerate community education about mental health and stress. From the project perspective, this digital approach for training and awareness-raising was affordable to develop and implement, and feasible for community members to use and share the films with others in their communities.

From a programmatic standpoint, Atmiyata can be more widely implemented in low-resource settings as it does not demand additional infrastructure or human resources. The foundation of the intervention is in utilizing resources already in communities and simultaneously strengthening existing public health linkages by developing working relationships and referral pathways with district-level and rural hospitals, primary health care centres and non-governmental organizations working in rural communities. In addition, community stakeholders are involved in the entire training and evaluation process, improving acceptability and scalability by encouraging local ownership over the program development process. Training community volunteers as local champions, and developing and using digital tools to enhance learning and feedback were low-cost components of the intervention program, which could be adapted for use in other low-resource contexts. Leveraging digital solutions is particularly attractive in countries like India given the mobile phone penetration rate in the country; there are over a billion mobile subscribers in India at present, and thus an easy to use app and community films can reach a larger population, regardless of location or social status, compared to face-to-face interventions. Other community-based interventions could consider using a central concept that holds high cultural acceptability in that particular context and design the intervention around this locally understood concept. In India, the concept of *Atmiyata* itself is highly regarded and accepted and the notion of shared compassion is embedded in the mentality of many Indians thus serving as a powerful central tenant of an intervention focused on improving community wellbeing. Finally, from a human resource perspective, given the volume of community groups like self-help groups and farmers’ clubs in India, this community platform may be a viable entry point for working at community level in other districts not only in India but in other parts of South Asia as well where such community groups also exist.

Scaling up and sustainability of *Atmiyata* is dependent on availability of ongoing supervision and support for *Atmiyata* champions. Field supervisors are important to appoint and need to work with community volunteers on an ongoing basis for addressing changes in motivation, troubleshooting and helping with using referral pathways. Funding is therefore required for an ongoing supervision and mentoring mechanism as well as for training new groups of Atmiyata champions. Although *Atmiyata* Champions currently work on a voluntary basis, in the long term and at scaled up level, it is possible that the volunteers may expect to be remunerated for their work similar to incentives paid to Accredited Social Health Activists (ASHA) workers. ASHA workers are an initiative of the Ministry of Health and Family Welfare of the Government of India which consist of community workers (1 per every 1000 people) who serve as the linking pin between community members and health care services, and receive incentives based on performance. Therefore, when scaling up the *Atmiyata* program, it is essential to inculcate the philosophy of shared compassion (*Atmiyata*) and the role of volunteering for greater community good.

## Abbreviations

CMD: common mental disorder
FC: farmer’s club
PHC: Primary Health Centre
SHG: self-help group
